# The Controlled Preparation of a Carrier-Free Nanoparticulate Formulation Composed of Curcumin and Piperine Using High-Gravity Technology

**DOI:** 10.3390/pharmaceutics16060808

**Published:** 2024-06-14

**Authors:** Ning Han, Yue Liu, Xin Liu, Pengyue Li, Yang Lu, Shouying Du, Kai Wu

**Affiliations:** School of Chinese Materia Medica, Beijing University of Chinese Medicine, Beijing 102488, China; hanning@bucm.edu.cn (N.H.); liuyuetcm@bucm.edu.cn (Y.L.); liuxin1011@bucm.edu.cn (X.L.); pengyuelee@bucm.edu.cn (P.L.); 700353@bucm.edu.cn (Y.L.)

**Keywords:** carrier-free nanoparticulate formulation, high-gravity technology, multidrug co-assemble nanoparticle, insoluble drug, bioavailability

## Abstract

Carrier-free nanoparticulate formulations are an advantageous platform for the oral administration of insoluble drugs with the expectation of improving their bioavailability. However, the key limitation of exploiting carrier-free nanoparticulate formulations is the controlled preparation of drug nanoparticles on the basis of rational prescription design. In the following study, we used curcumin (Cur) and piperine (Pip) as model water-insoluble drugs and developed a new method for the controlled preparation of carrier-free drug nanoparticles via multidrug co-assembly in a high-gravity environment. Encouraged by the controlled regulation of the nucleation and crystal growth rate of high-gravity technology accomplished by a rotating packed bed, co-amorphous Cur-Pip co-assembled multidrug nanoparticles with a uniform particle size of 130 nm were successfully prepared, exhibiting significantly enhanced dissolution performance and in vitro cytotoxicity. Moreover, the hydrogen bonding interactions between Cur and Pip in nanoparticles provide them with excellent re-dispersibility and storage stability. Moreover, the oral bioavailability of Cur was dramatically enhanced as a result of the smaller particle size of the co-assembled nanoparticles and the effective metabolic inhibitory effect of Pip. The present study provides a controlled approach to preparing a carrier-free nanoparticulate formulation through a multidrug co-assembly process in the high-gravity field to improve the oral bioavailability of insoluble drugs.

## 1. Introduction

The limitation of drugs with low water solubility in terms of dissolution behavior and oral bioavailability is a primary concern in the pharmaceutical field [1,2]. Despite the significant progress made in developing diverse drug delivery systems (DDSs), e.g., inorganic strategies, lipid-based nanoparticles, and polymer-based micelles, the complicated synthetic process, unsatisfactory drug loading capacity, and carrier-induced systemic toxicity still seriously impede their clinical application [3,4,5,6]. To overcome these obstacles, carrier-free nanoparticulate formulations (CNFs), formulated by reducing the particle size of active pharmaceutical ingredients (APIs) to the nanometer scale using a small amount of surfactant as a stabilizer, have emerged as an effective approach based on the Noyes–Whitney equation and Ostwald–Freundlich theory [7,8,9]. Besides improved dissolution performance, the better adhesion to biological membranes, an altered intestinal absorption mechanism, and the potential for targeted delivery are also considered important elements of CNFs in enhancing the bioavailability of insoluble drugs administered orally [10,11,12].

At present, the established key techniques for CNFs preparation can be mainly categorized into bottom-up technologies by controlling the precipitation process, and top-down technologies, which involve the employment of a high force to reduce particle size through grinding and high-pressure homogenization [13,14,15]. Despite the successful application of top-down technologies to commercial CNFs, there is a heightened risk of product contamination and degradation during the protracted processing stage [16,17]. More importantly, the poor efficiency of particle size reduction in the preparation of CNFs with a particle size <150 nm hampers the widespread development of top-down technologies [13]. Concurrently, the strict demands on nucleation and growth kinetics during the precipitation process, e.g., the nucleation rate of particles must surpass the growth rate at a uniform supersaturation level, also greatly increase the difficulty in achieving the controlled preparation of CNFs using bottom-up technologies [18]. For these reasons, most studies on CNFs focus on the efficient reduction in API particle size via prescription screening and the development of preparation technology but pay less attention to the comprehensive role of the formulation in in vivo performance [19,20]. Furthermore, nanoparticles assembled using different components have been demonstrated to have outstanding advantages as drug delivery systems in inhibiting system metabolization, avoiding multidrug resistance, and achieving synergistic therapy [21,22,23,24]. Therefore, the realization of the controlled preparation of a CNF containing multiple drug components could represent a promising direction for the oral administration of insoluble drugs.

The use of high-gravity technology, which relies on the artificial creation of a high-gravity environment using a rotating packed bed (RPB) and involves an acceleration range one to three orders of magnitude greater than the Earth’s gravitational acceleration, is regarded as an excellent intensification method for the precipitation process owing to its outstanding performance in strengthening molecular mixing and the mass transfer process [25,26]. In particular, the fluid in the RPB can be shredded into exceedingly minute droplets, threads, and thin films using the enormous shear force of a porous packed bed to fulfill the uniform concentration distribution in the RPB, thereby effectively impeding Ostwald ripening by narrowing the particle size distribution of the precipitated particle and effectively reducing the particle size of the drug nanoparticle [27]. Moreover, the findings from our previous research have confirmed that the nucleation rate of insoluble drugs in the RPB could be evidently accelerated such that the growth rate would be neglected, which efficiently facilitates the controlled preparation of ultrafine drug nanoparticles [28]. In light of the above reasons, high-gravity technology shows great potential in inducing enhanced particle size and size distribution in contrast with other bottom-up methods employed for the fabrication of CNFs. At present, the application of high-gravity technology is being extensively explored in the precipitation process of insoluble drugs, such as irbesartan, dasatinib, sirolimus, and cefixime, demonstrating that high-gravity technology is an invaluable platform to realize the controlled preparation of insoluble drug CNFs [29,30,31,32]. However, no study has explored the controlled preparation of CNFs containing different drugs through the use of high-gravity technology.

The primary objective of the following study is to realize the controlled preparation of an insoluble multidrug CNF by utilizing the regulation ability of high-gravity technology on the particle size in the liquid assembly process. Curcumin (Cur), as the active compound isolated from turmeric, exhibits various pharmacological effects, i.e., antitumor, anti-inflammatory, antivirus, and antioxidative properties [33,34,35]. However, the clinical application of Cur is hindered by its low water solubility, rapid metabolism, and poor membrane permeability [36,37]. In previous studies, it has been confirmed that the co-administration of Cur and piperine (Pip), a natural metabolic inhibitor, could significantly enhance the oral bioavailability of Cur by suppressing UGT enzymes, p450 enzymes, and the P glycoprotein efflux pump [38,39,40]. Nevertheless, the extent of enhancement in bioavailability for the Cur-Pip co-delivery system is also restricted by the poor water solubility of Pip [41,42]. In this regard, Cur and Pip were taken as the model drug pair in the present study, and the impact of RPB parameters on the particle size of Cur-Pip co-assembled nanoparticles was examined, followed by the characterization of solid-state properties. Furthermore, we also tested the physical stability, in vitro dissolution performance, and antitumor activity of the formulated Cur-Pip CNF. Lastly, the pharmacokinetic characteristics of the Cur-Pip CNF after oral administration were studied to examine whether the enhancement of Cur oral bioavailability could be achieved by the co-assembly of Cur and Pip as a CNF.

## 2. Materials and Methods

### 2.1. Materials

Of the main materials used in the present study, curcumin (>98%), was purchased from Shanghai Yien Chemical Technology Co., Ltd. (Shanghai, China), and piperine (>98%) was purchased from Baoji Fang Sheng Biological Development Co., Ltd. (Baoji, China). Tween-80, potassium phosphate monobasic, Dimethylsulfoxide (DMSO), and polyvinylpyrrolidone (PVP K30, MW = 58,000) were provided by Shanghai Macklin Biochemical Co., Ltd. (Shanghai, China). Methanol (MeOH, AR grade) was supplied by Beijing Tong Guang Fine Chemical Company (Beijing, China). Hydrochloric acid (HCl), sodium hydroxide, and ethyl acetate were purchased from Beijing Chemical Works (Beijing, China). Carboxymethyl cellulose sodium (CMC-Na) was procured from Tianjin Fuchen Chemical Reagents Factory (Tianjin, China). Verapamil was acquired from Shanghai yuanye Bio-Technology Co., Ltd. (Shanghai, China). 3-(4,5-dimethylthiazol-2-yl)-2,5-diphenyltetrazolium bromide (MTT) was purchased from Biodee Biotechnology Co., Ltd. (Beijing, China). Lastly, Tween-20 was provided by Shanghai Aladdin Biochemical Technology Co., Ltd. (Shanghai, China). Deionized water was used in all the experiments. Acetonitrile and formic and acetic acid purchased from Fisher Chemical (Shanghai, China) were all of chromatographic grade without any further purification.

### 2.2. Preparation of the Cur-Pip CNF

**Figure 1:**
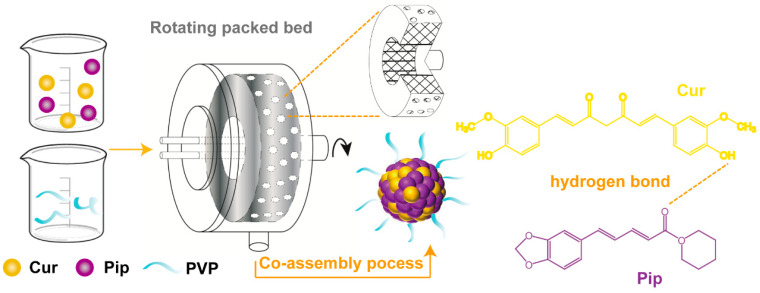
Schematic diagram for the co-assembly process of the Cur-Pip CNF in the RPB.

Figure 1 exhibits the Cur-Pip CNF prepared via the liquid co-assembly method using the RPB. Briefly, the solvent phase (Cur-Pip solution) was prepared by dissolving Cur (125 mg) and Pip (97 mg) into MeOH (50 mL), followed by filtration using 0.45 μm pore size membranes. The antisolvent phase (the PVP solution) was obtained by adding 222 mg PVP K30 into different volumes of deionized water (150, 400, 500, 550, and 600 mL) to facilitate the co-assembly process of Cur and Pip at different antisolvent/solvent ratios. In the co-assembly process, the Cur-Pip solution and the PVP solution were pumped together into the RPB at a constant flow rate of Cur-Pip solution (20 mL/min), and the Cur-Pip nanosuspension was immediately obtained at various rotating speeds. The detailed process parameters of the co-assembly process are listed in Table 1. The above formed Cur-Pip nanosuspension was collected from the outlet followed by 72 h lyophilization at a pressure of 6 Pa after freezing at −20 °C using a lyophilizer (Model FD-2A, Beijing Boyikang Experimental Instrument Co., Ltd., Beijing, China). Lastly, the dried Cur-Pip CNF (*W*_Cur_:*W*_Pip_:*W*_PVP_ = 1:0.77:1.77) was obtained and stored using a desiccator at room temperature before characterization and testing. As a comparison, the preparation of Cur-Pip CNF was also carried out in the stirred tank reactor (STR) through the addition of Cur-Pip solution into the PVP solution under vigorous stirring.

### 2.3. Characterization

The size and morphology of the Cur-Pip co-assembled nanoparticles were examined through the use of scanning electron microscopy (SEM, S4800, Hitachi, Tokyo, Japan). Nano Measurer 1.2 software was utilized to acquire particle size data in accordance with the SEM images. The crystal structure of the Cur-Pip CNF was analyzed through the use of X-ray diffraction (XRD), specifically with an Ultima IV X-ray powder diffractometer, which featured a scanning speed of 5°/min within the region of 5° to 40°, and the step size for this process was 0.02° (Rigaku, Tokyo, Japan). The thermal behavior of the Cur-Pip CNF was tested via differential scanning calorimetry (DSC, DSC 3, Mettler-Toledo, Zurich, Switzerland), utilizing a heating rate of 10 °C/min, spanning a temperature interval from 25 °C to 240 °C, within a nitrogen atmosphere (50 mL/min). The intermolecular interaction of Cur-Pip co-assembled nanoparticles was determined using the Fourier transform infrared (FTIR) spectra on an Alpha II spectrometer (Bruker, Luken, Germany) within the 400–4000 cm^−1^ spectral range.

### 2.4. In Vitro Dissolution Study

The dissolution properties of the Cur raw drug, Pip raw drug, Cur-Pip physical mixture (Cur-Pip PM, *W*_Cur_: *W*_Pip_: *W*_PVP_ = 1: 0.77: 1.77), and Cur-Pip CNF were assessed in 900 mL of pH 1.2 HCl buffer (7.65 mL of hydrochloric acid added to 1000 mL of deionized water) and pH 6.8 phosphate buffer (6.805 g of potassium phosphate and 0.944 g of sodium hydroxide added to 1000 mL of deionized water), supplemented with 0.5% Tween-80 using the D-800LS dissolution apparatus (Tianda Tianfa Technology Co., Ltd., Tianjin, China) at a temperature of 37 ± 0.5 °C and a rotating speed of 100 rpm. For each sample, a quantity equivalent to 10 mg of Cur was added into the vessel, and a 5 mL sample was subsequently drawn at predetermined time intervals. Additionally, 5 mL of fresh medium was added to maintain the original 900 mL volume. The amount of released Cur and Pip was, respectively, measured at 425 and 342 nm through the use of high-performance liquid chromatography (HPLC, UltiMate 3000, Thermo, Waltham, MA, USA). The HPLC analysis was carried out through the utilization of a Reprosil-Pur Basic C18 column (5 μm, 250 mm × 4.6 mm, Dr Maisch GmbH, Ammerbuch, Germany) maintained at a temperature of 30 °C. The mobile phase (a 50:50 *v*/*v* mixture of 4% acetic acid solution and acetonitrile) was isostatically pumped at 1 mL/min. All dissolution experiments were conducted in triplicate.

### 2.5. In Vitro Cytotoxicity Study

The 4T1 cells obtained from the ATCC (Manassas, VA, USA) were incubated in Roswell Park Memorial Institute-1640 medium (Mediatech, Inc., Manassas, VA, USA), supplemented with 10% fetal bovine serum (FBS, Gemini Biotechnology Co., Ltd., West Sacramento, CA, USA) and 1% penicillin–streptomycin (Life Technologies Corporation, Carlsbad, CA, USA). The incubation process took place within a humidified atmosphere containing 5% CO_2_ at a temperature of 37 °C. The cytotoxicity of the Cur raw drug, Cur-Pip PM, and Cur-Pip CNF on 4T1 cells was evaluated via the MTT assay. Briefly, the 4T1 cells were cultured in 96-well plates at a cell density of 10,000 per well. Following an overnight growth period, the cells were treated with the Cur raw drug, Cur-Pip PM, and Cur-Pip CNF dispersed in PBS buffer at predetermined drug levels and incubated over 24 and 48 h. After substitution of the drug-containing medium with fresh culture medium, MTT solution (10 μL, 5 mg/mL) was subsequently added to each well, with this step being followed by an incubation period of 4 h. Lastly, the supernatant was meticulously removed, and the formazan precipitated in each well was dissolved with 150 μL of DMSO. Absorbance was determined using a plate reader at 490 nm. Cell viability (%) was determined by comparing the absorbance of each well to that of the control wells, which solely contained the cell culture medium.

### 2.6. In Vivo Pharmacokinetics Study

The pharmacokinetic properties of the Cur raw drug, Pip raw drug, Cur-Pip PM, and Cur-Pip CNF were determined using male Sprague Dawley rats (with a weight of 250–260 g) supplied by SPF (Beijing, China) Biotechnology CO., Ltd. In a typical experiment, twenty-four rats were fasted overnight, given ad libitum access to water, and randomly assigned to four groups before drug administration. The Cur raw drug, Pip raw drug, Cur-Pip PM, and Cur-Pip CNF, dispersed in 0.5% CMC-Na and 0.1% Tween-20 solution, were administered by oral gavage with equivalent dosages of 150 mg/kg of Cur or 116 mg/kg of Pip. Blood samples were obtained at 0.083, 0.25, 0.5, 1, 2, 4, 6, 8, 12, and 24 h post-dosing via the orbital vein into heparinized Eppendorf tubes and then subjected to centrifugation at 4000 rpm for 8 min. The resulting plasma was subsequently transferred into sealed tubes and preserved at a temperature of −20 °C until further analysis.

#### 2.6.1. Preparation of Samples for Analysis

A volume of 250 μL from each thawed plasma sample was placed in microcentrifuge tubes, and 10 μL of verapamil dissolved in MeOH was then introduced as an internal standard. Subsequently, the mixture was mixed with 1 mL of ethyl acetate and subjected to vortexing for 5 min to extract the substances. Next, 800 μL of extract was transferred from 1 mL of the upper organic layer to a clean tube subjected to centrifugation and evaporation. Lastly, the remaining residue was reconstituted using 100 μL of MeOH and analyzed using an LC-MS/MS system consisting of a Q-Exactive HF-X mass spectrometer (Thermo, Waltham, MA, USA) and an UltiMate 3000 HPLC (Thermo, Waltham, MA, USA) equipped with an electrospray ionization source. An Accucore^TM^ C18 column (2.6 μm, 100 mm × 2.1 mm, Thermo, Waltham, MA, USA) was adopted for sample isolation, employing a mobile phase consisting of 0.1% formic acid aqueous solution (A) and 0.1% formic acid acetonitrile solution (B) at a flow rate of 0.3 mL/min. The gradient procedure was established as follows: 0–2 min: 5% B, 2–7 min: 5–45% B, 7–12 min: 45–55% B, 12–13 min: 55–95% B, 13–16 min 95%B, 16–17 min 95–5% B, 17–22 min 5% B. Quantification was performed using the transitions of m/z CUR: 369.1331/285.1114 (collision energy: 18 V), PIP: 286.1434/201.0545 (collision energy: 22 V), and verapamil: 455.2888/165.0907 (collision energy: 30 V).

#### 2.6.2. Determination of Pharmacokinetic Parameters

The pharmacokinetic parameters, including the area under the plasma concentration–time curve (AUC), the maximum plasma concentration (C_max_), and the time required to reach the Cmax (t_max_), were directly obtained from the plasma concentration versus time profiles, utilizing a non-compartmentalized methodology implemented in DAS 2.0.

### 2.7. Statistical Analysis

All data were presented as means ± SD. Student’s t-test or an ANOVA were employed for statistical analysis. Values with *p* < 0.05 were regarded as statistically significant.

## 3. Results and Discussion

### 3.1. Influence of Temperature on Particle Size

**Figure 2:**
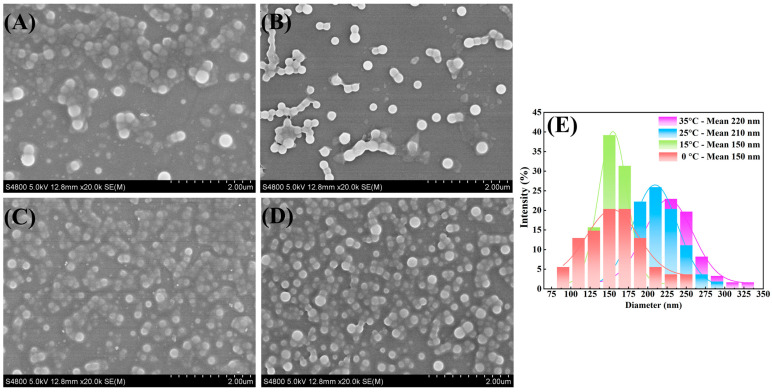
(**A**–**D**) SEM images of Cur-Pip nanoparticles assembled under different temperatures: (**A**) 35 °C, (**B**) 25 °C, (**C**) 15 °C, (**D**) 0 °C, and (**E**) the corresponding particle size distributions.

The preparation of ultrafine nanoparticles via precipitation is primarily dependent on the controlled regulation of the particle nucleation and growth process. According to the nucleation and growth rate equation, the assembly temperature is regarded as an important parameter affecting the particle size of the nanoparticle. In order to examine the effect of temperature on the particle size of multidrug co-assembly nanoparticles, Cur and Pip were allowed to assemble at different temperatures by fixing the antisolvent/solvent ratio at 11/1 and the rotating speed at 600 rpm. As displayed in Figure 2, it was noted that the particle size of the Cur-Pip nanoparticle diminished progressively from 220 nm to 150 nm, corresponding to a reduction in temperature from 35 to 15 °C. This result could be due to the fact that the reduction in temperature resulted in a faster nucleation rate and a relatively slower growth rate, making the particle size effectively decrease. Additionally, compared with the nucleation rate, the growth rate could be neglected with the sharp increase caused by the continuous reduction in temperature, which ensured the preparation of uniform nanoparticles with a smaller particle size. However, when the temperature was further decreased to 0 °C, the particle size distribution of the Cur-Pip nanoparticles broadened with an unchanged particle size of 150 nm. It is worth noting that the smaller particle size may lead to ease of agglomeration, thereby inducing a broader particle size distribution at lower temperatures.

### 3.2. Influence of Antisolvent/Solvent Ratio on Particle Size

**Figure 3:**
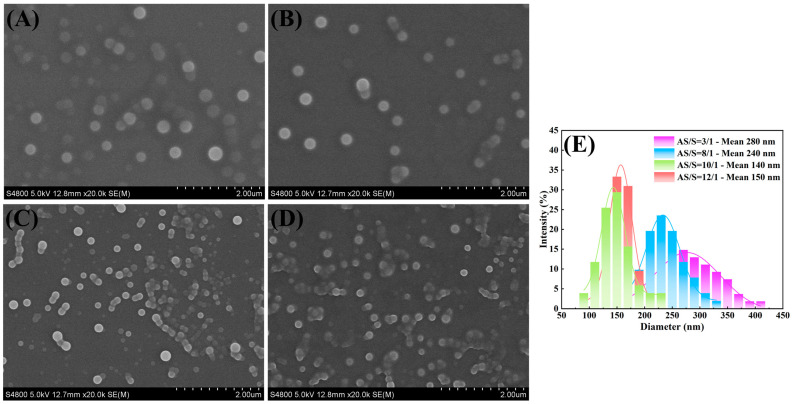
(**A**–**D**) SEM images of Cur-Pip nanoparticles assembled under various AS/S flow ratios: (**A**) 3/1, (**B**) 8/1, (**C**) 10/1, and (**D**) 12/1 and (**E**) the corresponding particle size distributions.

The supersaturation level of the system, caused by the mixing of the solvent (S) and antisolvent (AS), is the driving force for the preparation of nanoparticles via precipitation. As such, the regulation of the AS/S ratio is vital for the liquid co-assembly process. As shown in Figure 3, it could be discerned that the size of Cur-Pip nanoparticles decreased from 280 nm to 140 nm as the AS/S ratio increased from 3:1 to 10:1. However, a marginal increase in the average particle size could be detected when the AS/S ratio continued to increase to 12:1. The following causes could potentially account for this phenomenon. Firstly, with the increase in the AS/S ratio from 3:1 to 10:1, the higher level of supersaturation could effectively enhance the relative rate ratio of the nucleation and growth process by dramatically accelerating the nucleation process, making the particle size of Cur-Pip nanoparticles significantly decrease. Secondly, as the AS/S ratio continued to increase, the nucleation and growth rate remained practically unchanged owing to the constant supersaturation level, as demonstrated in our previous study [28]. Consequently, the nanoparticles maintained a smaller particle size. However, as the AS flow rate increased, the concentration of stabilizer decreased, resulting in the growth of nanoparticles through agglomeration. Accordingly, the particle size of Cur-Pip nanoparticles decreased and then increased when the AS/S ratio increased.

### 3.3. Influence of RPB Rotating Speed on Particle Size

**Figure 4:**
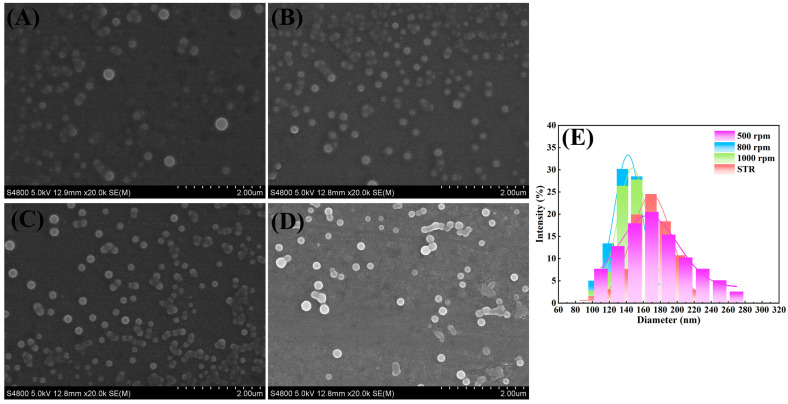
(**A**–**C**) SEM images of Cur-Pip nanoparticles assembled in the RPB at different rotating speeds: (**A**) 500 rpm, (**B**) 800 rpm, and (**C**) 100 rpm. (**D**) SEM image of Cur-Pip NPs prepared in the STR. (**E**) The corresponding particle size distributions.

The homogenization level of supersaturation before the nucleation process dramatically influences the particle size and particle size distribution of nanoparticles generated through the precipitation process. Specifically, the characteristic time of the micro-mixing (t_m_) of the precipitation reactor, reflecting the micro-mixing performance, has been proven to be typically less than 1 ms (the induction time of nucleation, τ). Since the intensification level of micro-mixing by the RPB is highly dependent on the rotating speed, the Cur-Pip nanoparticles were assembled at different rotating speeds in the RPB and STR to determine the influence of rotating speed on the particle size. According to Figure 4, the average particle size of the Cur-Pip nanoparticles decreased from 170 nm to 130 nm as the rotation speed increased from 500 rpm to 800 rpm. In addition, the particle size of the Cur-Pip nanoparticles remained around 170 nm when assembled in the STR under the same conditions. Therefore, compared to the STR, the micro-mixing performance in the RPB could actually be conducive to the preparation of nanoparticles with a small size. When the rotating speed of the RPB increased further to 1000 rpm, the particle size distribution observed for Cur-Pip NPs increased, with the particle size slightly increasing to 140 nm. Although it has been demonstrated that a faster rotating speed offers a higher nucleation rate owing to the increased molecular transport by the shear force of the packed rotator in our previous study, the agglomeration of nanoparticles can also occur due to the increased probability of collision between nanoparticles, contributing to the enlarged particle size and broadening of the particle size distribution.

### 3.4. Physicochemical Characterization of Cur-Pip CNF

**Figure 5:**
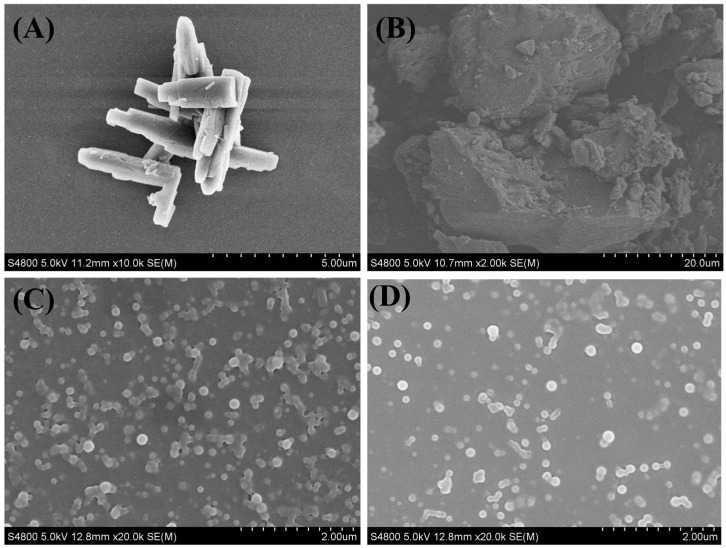
(**A**–**D**) SEM images of the Cur raw drug, Pip raw drug, redispersed Cur-Pip nanoparticles after freeze-drying, and redispersed Cur-Pip nanoparticles stored for 180 days.

The morphology of Cur raw drug, Pip raw drug, and Cur-Pip CNF was characterized by SEM. Cur raw drug (Figure 5A) and Pip raw drug (Figure 5B) showed rod-like shapes and irregular bulk-like shapes with rough surfaces, respectively. However, as exhibited in Figure 4B, Cur-Pip CNF was a uniform sphere in terms of shape with a particle size of around 130 nm, indicating that co-assembly nanotechnology may hold great promise as a strategic approach to improving the bioavailability of Cur and Pip by improving the dissolution process. In addition, the dried Cur-Pip nanoparticles were well redispersed in water, accompanied by minimal alteration in morphology and particle size (Figure 5C). The above finding suggests that Cur-Pip co-assembled nanoparticles could maintain their stability following freeze-drying thanks to the robust protection provided by the PVP and the intermolecular interaction between Cur and Pip. This hypothesis would need to be comprehensively confirmed using FTIR spectra.

**Figure 6:**
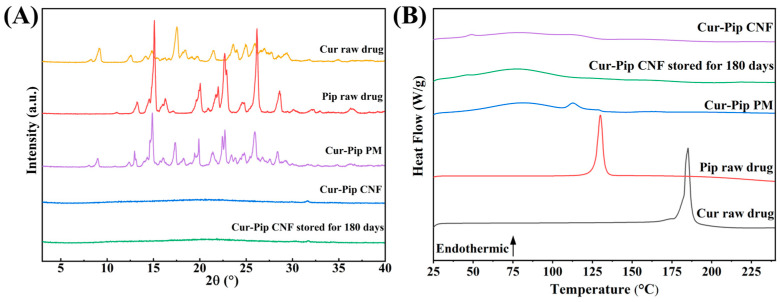
(**A**) XRD patterns of the Cur raw drug, Pip raw drug, Cur-Pip PM, Cur-Pip CNF, and Cur-Pip CNF stored for 180 days. (**B**) DSC curves of the Cur raw drug, Pip raw drug, Cur-Pip PM, Cur-Pip CNF, and Cur-Pip CNF stored for 180 days.

The XRD analysis was conducted to assess the crystalline structure of Cur and Pip within the Cur-Pip CNF. As presented in Figure 6A, the Cur raw drug and Pip raw drug exhibited multiple sharp and distinct peaks, indicating that both Cur and Pip raw drugs existed as crystalline structures. Additionally, the characteristic peaks from the Cur raw drug and Pip raw drug were maintained in the Cur-Pip PM. However, the distinctive peaks of Cur and Pip disappeared, and a broad diffuse peak was observed for the Cur-Pip CNF. Furthermore, the thermal behavior of the Cur-Pip CNF was studied via a DSC assay. As shown in Figure 6B, the DSC curves of the Cur raw drug and Pip raw drug showed distinct endothermic peaks around ~185 °C and ~130 °C, respectively, corresponding to the melting points of Cur and Pip. In comparison, in the Cur-Pip CNF, these endothermic peaks disappeared. Therefore, the XRD and DSC results jointly demonstrate that the Cur-Pip nanoparticles were in the co-amorphous form.

**Figure 7:**
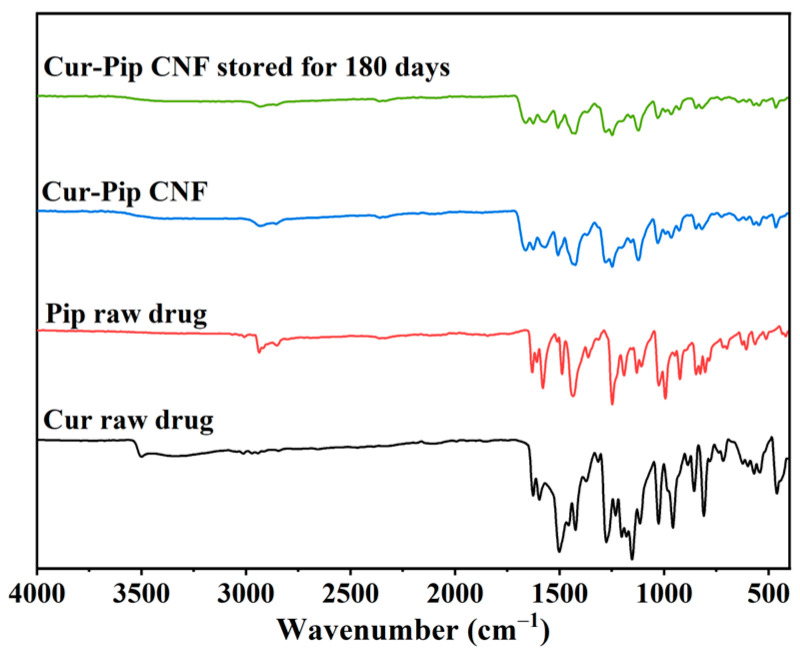
FTIR spectra of the Cur raw drug, Pip raw drug, Cur-Pip CNF, and Cur-Pip CNF stored for 180 days.

The molecular status of the Cur-Pip CNF was characterized using FTIR analysis to provide information about the interaction mechanism between Cur and Pip in the co-assembled nanoparticles. As shown in Figure 7, the stretching vibration peaks of the Cur raw drug at 3501 and 1626 cm^−1^ were attributed to phenolic hydroxyl and C=O groups. The peaks observed at 2934 cm^−1^ and 1632 cm^−1^ in the case of the Pip raw drug were attributed to the stretching vibrations of the C-H group of the benzene ring as well as the C=O group, respectively. For the co-amorphous Cur-Pip nanoparticles, the characteristic absorption peaks of the phenolic OH group (3501 cm^−1^) in Cur and the C=O group (1632 cm^−1^) in Pip disappeared or decreased, indicating that intermolecular hydrogen bonds formed between Cur and Pip. Therefore, hydrogen bonding between Cur and Pip could be the potential driving force behind the assembly of the co-amorphous system.

As stability is of great importance for amorphous nanoparticulate formulations in the high-energy solid state, the stability of the Cur-Pip CNF during the storage was monitored regarding particle size, morphology, crystalline form, and composite characteristics after storage at room temperature under dark conditions for 180 days. According to Figure 5D, the average particle size obtained from the redispersed Cur-Pip nanoparticles remained at 130 nm with uniform spherical morphology. The results of the XRD analysis (Figure 6A) indicated the Cur-Pip CNF stored for 180 days also remained amorphous, a finding that aligns with the results obtained from the DSC analysis (Figure 6B). In addition, the composite analysis of the Cur-Pip CNF following storage for 180 days demonstrated a satisfactory concurrence with the freshly prepared sample (Figure 7). The above findings imply that the assembled Cur-Pip nanoparticles were sufficiently stable due to the intermolecular interaction of hydrogen bonding and the protection provided by the stabilizer PVP.

### 3.5. In Vitro Dissolution Study

**Figure 8:**
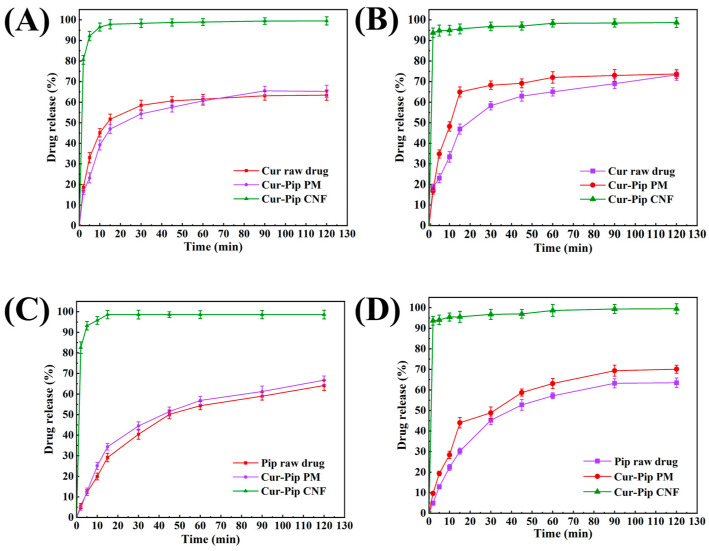
In vitro dissolution profiles of Cur in (**A**) HCl buffer (pH = 1.2) and (**B**) phosphate buffer (pH = 6.8). In vitro dissolution profiles of Pip in (**C**) HCl buffer (pH = 1.2) and (**D**) phosphate buffer (pH = 6.8).

Dissolution performance is regarded as a vital method in predicting the in vivo behavior. The release performance of the Cur-Pip CNF was evaluated under various pH conditions with the Cur raw drug, Pip raw drug, and Cur-Pip PM at the equivalent Cur-Pip CNF dosage as controls. As shown in Figure 8, approximately only 60% of Cur and 55% of Pip were released from the corresponding raw drug at pH 6.8 as well as pH 1.2 HCl during the 2 h release period. Moreover, there was almost no change in the dissolution behavior regarding Cur and Pip for the physical mixture compared to the raw drug form, which implied that the stabilizer (PVP) and drug combination had nearly no influence on the dissolution performance and solubility of the active ingredients. However, the Cur-Pip CNF exhibited a significantly improved dissolution rate of Cur and Pip. The dissolution rate was found to be above 95% for Cur and Pip released from the CNF form at a pH of 1.2 or 6.8 over 2 min, indicating that the co-assembled nanoparticle might facilitate the enhancement of the bioavailability of Cur and Pip via the increased solubility owing to particle size reduction.

### 3.6. Cytotoxicity Study

**Figure 9:**
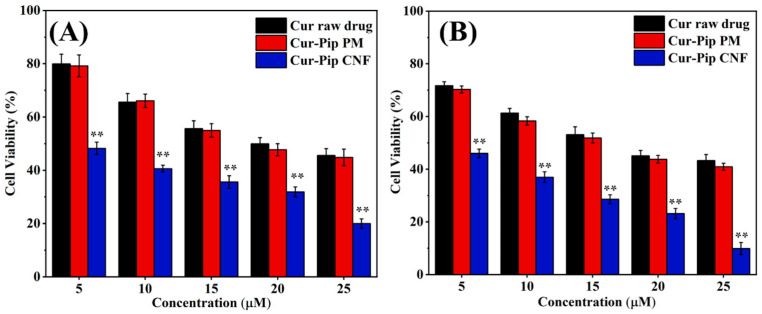
Concentration-dependent cytotoxicity of the Cur raw drug, Cur-Pip PM, and Cur-Pip CNF in 4T1 cells at (**A**) 24 h and (**B**) 48 h; **, *p* < 0.01 vs. the Cur raw drug.

Figure 9 shows the percentage of cell viability under various levels of Cur, the Cur-Pip PM, and the Cur-Pip CNF. In our experiments, we found no significant differences in antitumor activity for the Cur raw drug and the Cur-Pip PM, suggesting the unsatisfactory antitumor effect caused by the low dissolution rate of the raw drug with a larger particle size. It is noteworthy that Cur-Pip CNF inhibited 4T1 cell proliferation in a dose- and time-dependent manner. In addition, a dramatic decrease in cell survival rate was detected for the Cur-Pip CNF-treated cells compared to the Cur raw-drug-treated and Cur-Pip PM-treated cells under identical experimental conditions. Therefore, the antiproliferation activity of the Cur-Pip drug pair was significantly improved by the co-assembled nanoparticles due to the promotion of the drug dissolution process.

### 3.7. Pharmacokinetic Study

**Figure 10:**
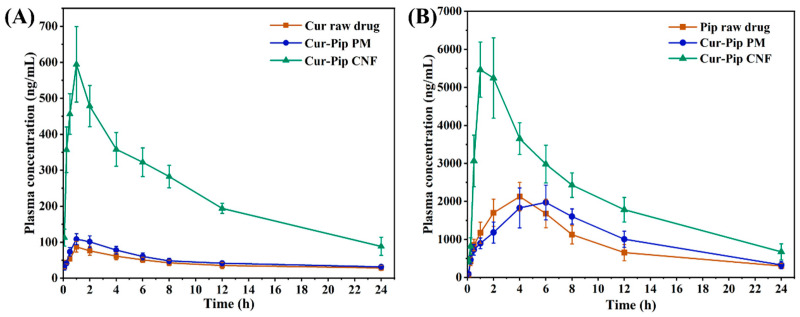
(**A**) Plasma drug concentration–time curve of Cur following the oral administration of the Cur raw drug, Cur-Pip PM, and Cur-Pip CNF equivalent to a dose of 150 mg/kg Cur. (**B**) Plasma drug concentration–time curve of Pip following the oral administration of the Pip raw drug, Cur-Pip PM, and Cur-Pip CNF equivalent to a dose of 116 mg/kg Pip.

The plasma concentration–time profiles of Cur and Pip following the oral administration of Cur raw drug, Pip raw drug, Cur-Pip PM, and Cur-Pip CNF are illustrated in Figure 10, in addition to the corresponding pharmacokinetic parameters listed in Table 2. As summarized in Table 2, although the C_max_ value of Cur was elevated by the co-administration of Cur and Pip as a physical mixture, no significant differences were found in the T_max_ and AUC_0–24h_ of Cur in comparison with the Cur raw drug. Moreover, in contrast with the Pip raw drug, there were no notable disparities in the T_max_, C_max_, and AUC_0–24h_ of Pip in the Cur-Pip PM. In comparison, significant increases were found in the C_max_ and AUC_0–24h_ of Cur and Pip after 24 h of oral administration following co-assembly by Cur and Pip into nanoparticles. In particular, the C_max_ values of Cur and Pip from the Cur-Pip CNF (601.27 ± 96.19 ng/mL and 6092.44 ± 402.59 ng/mL) were higher than those from the raw drug (90.40 ± 12.80 ng/mL and 2203.86 ± 248.33 ng/mL) and Cur-Pip PM (108.69 ± 15.20 ng/mL and 2141.58 ± 459.51 ng/mL). The AUC_0–24h_ value of Cur from the Cur-Pip CNF was 5713.5 ± 307.84 ng·h/mL, which was roughly 5.60 and 4.69 times that of the Cur raw drug and Cur from the Cur-Pip PM, respectively. Similarly, the AUC_0–24h_ of Pip from the Cur-Pip CNF increased by around 2.38 and 2.07 times that in the raw drug and Cur-Pip PM. In addition, the t_max_ of Pip following oral administration in the Cur-Pip CNF was shorter than that of the Pip raw drug and Cur-Pip PM. Therefore, the above findings demonstrate that the oral co-delivery of Cur and Pip by carrier-free co-assembled nanoparticles could significantly enhance the oral absorption of Cur. The underlying reasons could be ascribed to (1) the dissolution performance of Cur being greatly improved by the reduction in particle size and the co-amorphous form and (2) the simultaneously released Pip effectively inhibiting the metabolization of Cur.

## 4. Conclusions

In summary, carrier-free multidrug co-assembly nanoparticles were successfully prepared in the study described herein using high-gravity technology with Cur and Pip utilized as a model drug pair, providing a prospective avenue for the fabrication of a multidrug CNF with uniform particle size and preferable bioavailability. The as-prepared Cur-Pip nanoparticles were in a co-amorphous state with an average size of approximately 130 nm under optimal conditions. Moreover, the Cur-Pip CNF exhibited outstanding re-dispersibility and storage stability due to the interaction between Cur and Pip, mediated by intermolecular hydrogen bonding. The dissolution rate and in vitro cytotoxicity of the Cur-Pip CNF were significantly enhanced compared with the coarse drug and physical mixture. Lastly, the enhancement of Cur bioavailability was notably achieved through the reduction in particle size and the metabolic inhibition of the released Pip. The results presented herein demonstrate that the controlled preparation of carrier-free multidrug nanoparticles using an RPB is a promising method for enhancing the oral bioavailability of insoluble drugs.

## Figures and Tables

**Table 1:** The process parameters of high-gravity technology and the average particle size of Cur-Pip nanoparticles prepared under the corresponding process parameters.

Process Parameters of High-Gravity Technology			Particle Size of Cur-Pip Nanoparticles (nm)
Temperature (°C)	AS/S Ratio	Rotating Speed of RPB (rpm)	
35	11/1	600	220
25	11/1	600	210
15	11/1	600	150
0	11/1	600	150
15	3/1	600	280
15	8/1	600	240
15	10/1	600	140
15	12/1	600	150
15	10/1	500	170
15	10/1	800	130
15	10/1	1000	140

**Table 2:** Pharmacokinetic parameters of drugs in the rats following the oral administration of the Cur raw drug, Pip raw drug, Cur-Pip PM, and Cur-Pip CNF.

Model Drug	Parameters	Raw Drug	Cur-Pip PM	Cur-Pip CNF
Cur	C_max_ (ng/mL)	90.40 ± 12.80	108.69 ± 15.20 *	601.27 ± 96.19 **
	T_max_ (h)	1.33 ± 0.52	1 ± 0	1.17 ± 0.41
	AUC_0–24 h_ (ng·h/mL)	1019.76 ± 168.33	1217.19 ± 128.59	5713.5 ± 307.84 **
Pip	C_max_ (ng/mL)	2203.86 ± 248.33	2141.58 ± 459.51	6092.44 ± 402.59 ^##^
	T_max_ (h)	3.67 ± 0.82	5 ± 1.10	1.33 ± 0.52 ^##^
	AUC_0–24_ h (ng·h/mL)	21,894.12 ± 2790.69	25,259.18 ± 4006.03	52,162.70 ± 4812.74 ^##^

*, *p* < 0.05; **, *p* < 0.01, vs. Cur raw drug. ^##^, *p* < 0.01 vs. the Pip raw drug.

## Data Availability

Data are contained within the article and Supplementary Materials.

## Supplementary Materials

The following supporting information can be downloaded at: https://www.mdpi.com/article/10.3390/pharmaceutics16060808/s1, Figure S1. (A–D) The original SEM images of Cur-Pip nanoparticles assembled under different temperatures: (A) 35 °C, (B) 25 °C, (C) 15 °C, (D) 0 °C. Figure S2. (A–D) The original SEM images of Cur-Pip nanoparticles assembled under various AS/S flow ratios: (A) 3/1, (B) 8/1, (C) 10/1, and (D) 12/1. Figure S3. (A–C) The original SEM images of Cur-Pip nanoparticles assembled in the RPB at different rotating speeds: (A) 500 rpm, (B) 800 rpm, and (C) 100 rpm. (D) The original SEM image of Cur-Pip NPs prepared in the STR. Figure S4. (A–D) The original SEM image of the Cur raw drug, Pip raw drug, redispersed Cur-Pip nanoparticles after freeze-drying, and redispersed Cur-Pip nanoparticles stored for 180 days. Table S1. Plasma drug concentration of Cur following the oral administration of the Cur raw drug at a dose of 150 mg/kg. Table S2. Plasma drug concentration of Cur following the oral administration of the Cur-Pip PM equivalent to a dose of 150 mg/kg Cur. Table S3. Plasma drug concentration of Cur following the oral administration of the Cur-Pip CNF equivalent to a dose of 150 mg/kg Cur. Table S4. Plasma drug concentration of Pip following the oral administration of the Pip raw drug at a dose of 116 mg/kg. Table S5. Plasma drug concentration of Pip following the oral administration of the Cur-Pip PM equivalent to a dose of 116 mg/kg Pip. Table S6. Plasma drug concentration of Pip following the oral administration of the Cur-Pip CNF equivalent to a dose of 116 mg/kg Pip.
