# Supplementary material for: The Controlled Preparation of a Carrier-Free Nanoparticulate Formulation Composed of Curcumin and Piperine Using High-Gravity Technology
Source: Pharmaceutics. 2024 Jun 14;16(6):808. doi: 10.3390/pharmaceutics16060808 (PMC11207529; doi:10.3390/pharmaceutics16060808)
Supplement: Supplementary file 1 [file pharmaceutics-16-00808-s001.zip › pharmaceutics-2981965-supplementary.pdf]

The Controlled Preparation of a Carrier-Free Nanoparticulate Formulation Composed of Curcumin and Piperine Using High-Gravity Technology

Ning Han †, Yue Liu †, Xin Liu, Pengyue Li, Yang Lu, Shouying Du * and Kai Wu *

School of Chinese Materia Medica, Beijing University of Chinese Medicine, Beijing 102488, China; hanning@bucm.edu.cn (N.H.); liuyuetcm@bucm.edu.cn (Y.L.); liuxin1011@bucm.edu.cn (X.L.); pengyuelee@bucm.edu.cn (P.L.); 700353@bucm.edu.cn (Y.L.)

* Correspondence: 602066@bucm.edu.cn (S.D.); wukai@bucm.edu.cn (K.W.)

† These authors contributed equally to this work.

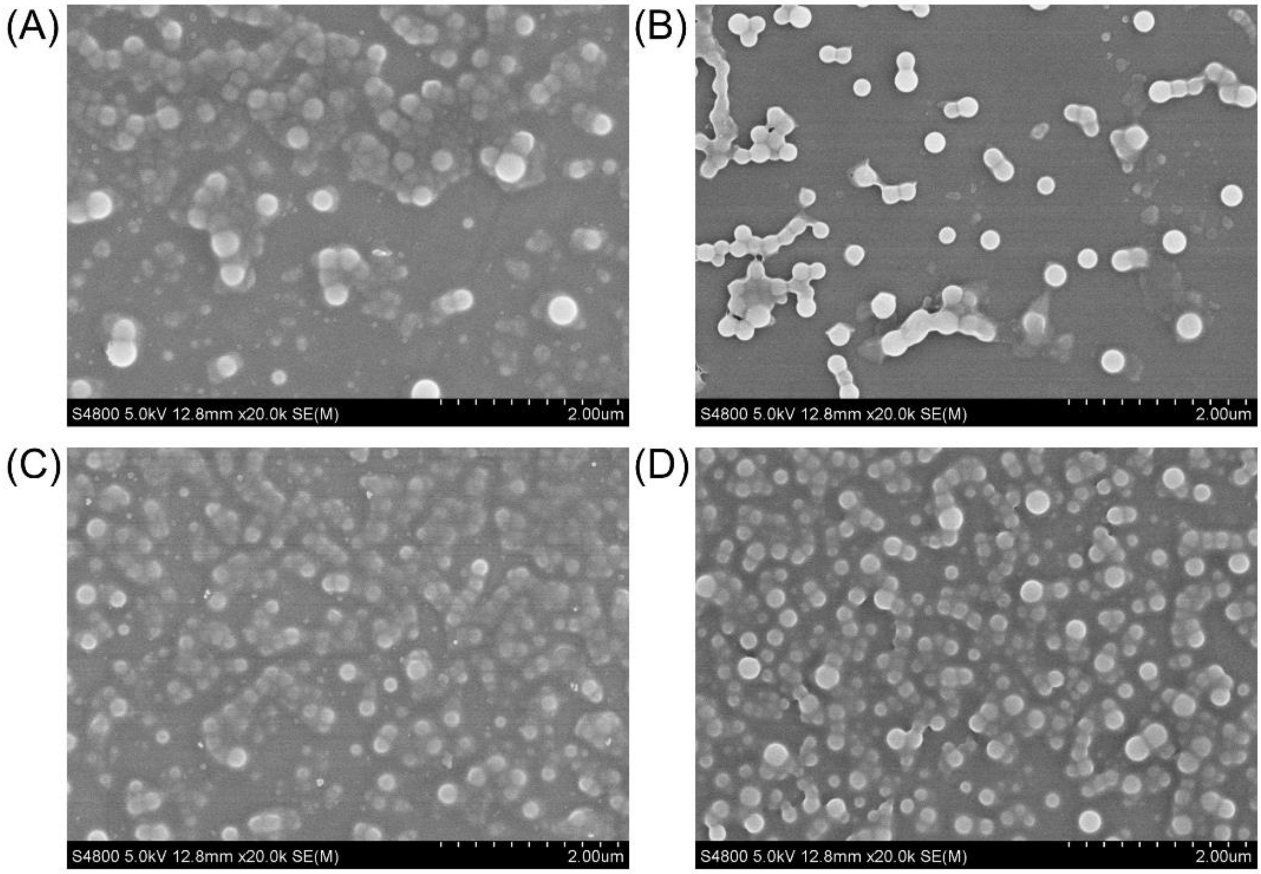

Figure S1. (A–D) The original SEM images of Cur-Pip nanoparticles assembled under different temperatures: (A) 35 °C, (B) 25 °C, (C) 15 °C, (D) 0 °C.

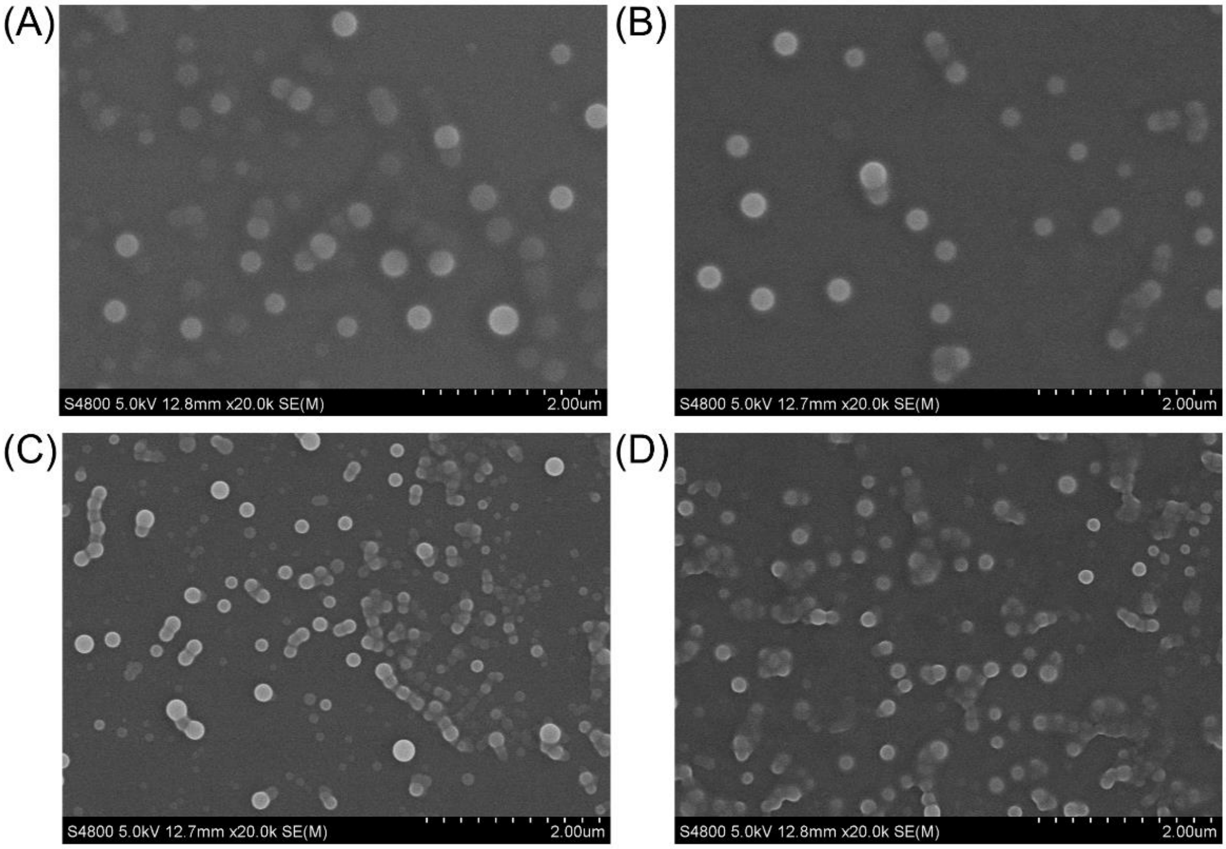

Figure S2. (A–D) The original SEM images of Cur-Pip nanoparticles assembled under various AS/S flow ratios: (A) 3/1, (B) 8/1, (C) 10/1, and (D) 12/1.

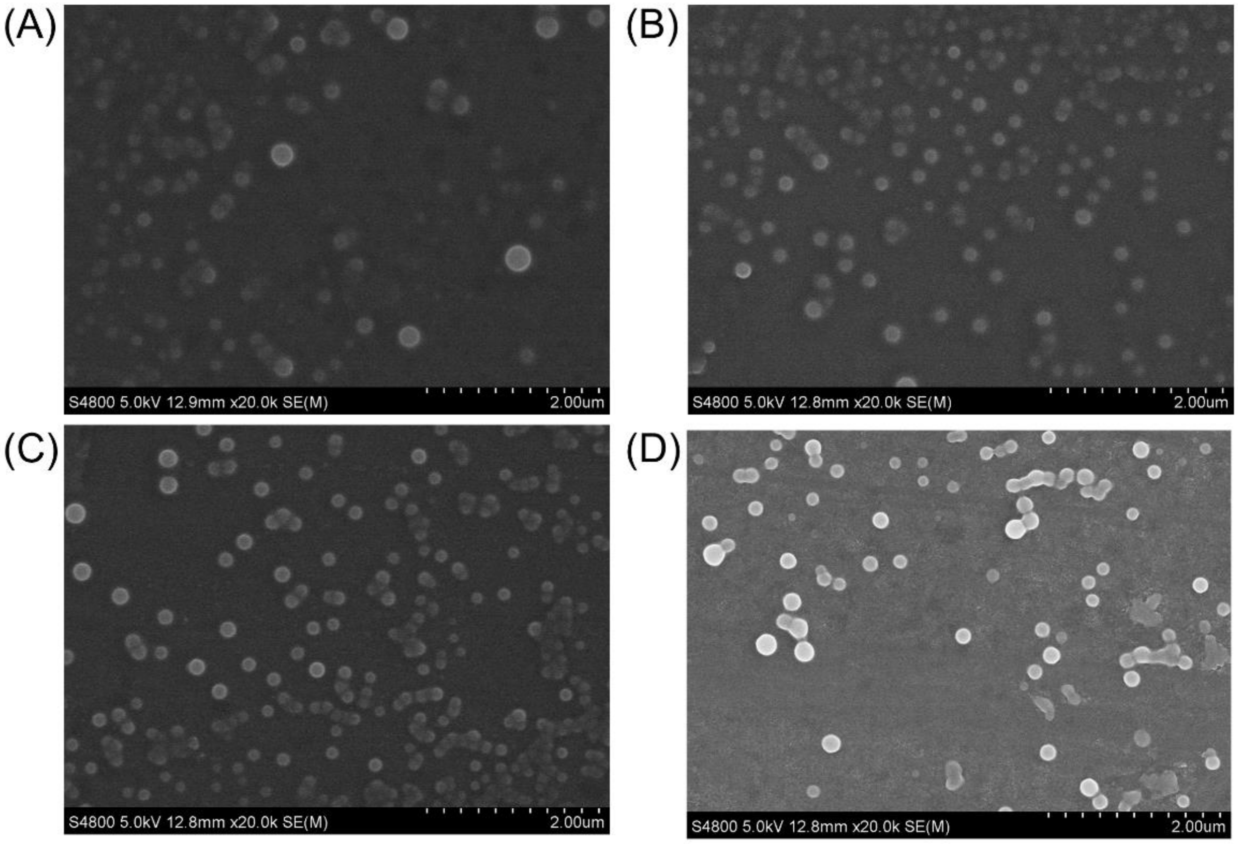

Figure S3. (A–C) The original SEM images of Cur-Pip nanoparticles assembled in the RPB at different rotating speeds: (A) 500 rpm, (B) 800 rpm, and (C) 100 rpm. (D) The original SEM image of Cur-Pip NPs prepared in the STR.

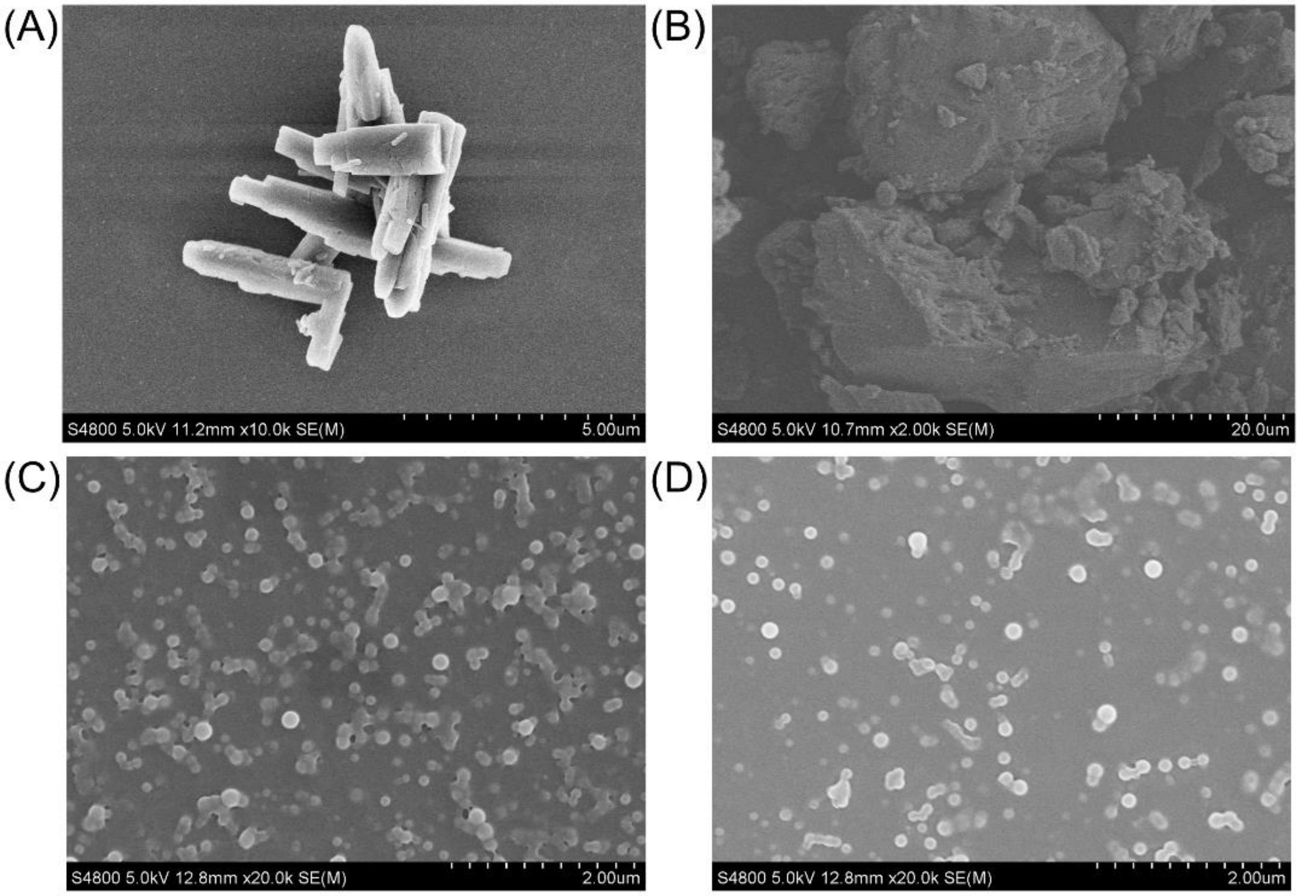

Figure S4. (A–D) The original SEM image of the Cur raw drug, Pip raw drug, redispersed Cur-Pip nanoparticles after freeze-drying, and redispersed Cur-Pip nanoparticles stored for 180 days.

Table S1. Plasma drug concentration of Cur following the oral administration of the Cur raw drug at a dose of 150 mg/kg.

**Time (h)**	**Plasma drug concentration (ng/mL)**					
	**Rat 1**	**Rat 2**	**Rat 3**	**Rat 4**	**Rat 5**	**Rat 6**
0.083	26.25	31.85	27.82	32.73	41.92	31.23
0.25	45.32	47.49	37.53	48.08	47.74	38.97
0.5	54.36	55.02	58.01	57.04	54.79	42.92
1	65.93	91.25	79.00	81.63	106.78	98.76
2	70.54	72.75	93.47	79.22	83.42	57.64
4	52.57	68.74	74.39	60.21	62.97	48.40
6	44.63	47.95	62.83	49.73	56.25	44.95
8	39.33	37.79	42.43	46.49	55.51	34.35
12	26.87	30.64	35.57	39.44	49.72	29.87
24	20.47	24.37	29.67	32.72	39.84	24.35

Table S2. Plasma drug concentration of Cur following the oral administration of the Cur-Pip PM equivalent to a dose of 150 mg/kg Cur.

Time (h)	Plasma drug concentration (ng/mL)					
	**Rat 1**	**Rat 2**	**Rat 3**	**Rat 4**	**Rat 5**	**Rat 6**
0.083	21.01	37.42	35.60	24.31	38.66	24.35
0.25	37.84	48.21	38.40	48.63	41.33	34.60
0.5	77.59	64.31	93.23	66.75	77.25	61.05
1	94.68	117.64	133.25	92.56	104.78	108.74
2	87.40	104.34	130.16	85.13	98.73	102.17
4	65.37	70.14	75.32	77.23	91.11	89.70
6	57.23	66.20	54.29	49.33	76.25	59.56
8	46.16	53.24	47.82	37.01	56.61	46.74
12	35.37	41.13	40.17	35.21	46.82	50.80
24	24.60	36.27	29.88	30.33	27.91	41.13

Table S3. Plasma drug concentration of Cur following the oral administration of the Cur-Pip CNF equivalent to a dose of 150 mg/kg Cur.

Time (h)	Plasma drug concentration (ng/mL)					
	**Rat 1**	**Rat 2**	**Rat 3**	**Rat 4**	**Rat 5**	**Rat 6**
0.083	147.74	117.39	106.37	80.56	97.98	126.61
0.25	337.69	407.38	313.02	455.67	344.03	284.04
0.5	526.53	503.63	365.96	457.84	452.33	432.59
1	604.74	784.88	564.59	539.36	598.50	473.90
2	515.55	477.74	533.54	448.91	379.82	515.54
4	374.32	411.47	306.47	410.79	326.70	319.09
6	344.57	360.44	266.66	364.55	303.19	295.08
8	240.38	331.50	263.90	300.40	282.91	275.83
12	174.50	196.25	198.21	181.27	199.68	213.87
24	105.78	80.46	97.77	120.65	78.47	48.06

Table S4. Plasma drug concentration of Pip following the oral administration of the Pip raw drug at a dose of 116 mg/kg.

Time (h)	Plasma drug concentration (ng/mL)					
	**Rat 1**	**Rat 2**	**Rat 3**	**Rat 4**	**Rat 5**	**Rat 6**
0.083	16.04	58.38	84.50	81.89	79.82	92.89
0.25	297.50	308.03	518.92	370.48	510.10	551.90
0.5	810.83	765.83	1117.77	902.59	855.17	671.94
1	970.00	1334.36	1675.12	1035.26	1002.08	1033.26
2	1281.48	1240.13	2096.07	1977.29	1780.60	1831.14
4	2050.78	2255.37	2354.68	1514.73	2602.72	1982.32
6	975.97	1984.43	1925.02	1559.58	1839.56	1807.61
8	827.30	1546.36	1010.93	1032.59	1117.63	1210.53
12	492.06	591.99	671.69	775.92	992.55	401.12
24	374.09	351.44	212.39	244.28	296.38	342.46

Table S5. Plasma drug concentration of Pip following the oral administration of the Cur-Pip PM equivalent to a dose of 116 mg/kg Pip.

Time (h)	Plasma drug concentration (ng/mL)					
	**Rat 1**	**Rat 2**	**Rat 3**	**Rat 4**	**Rat 5**	**Rat 6**
0.083	70.35	53.41	104.19	100.27	115.81	90.99
0.25	368.00	387.43	388.97	482.76	661.38	510.46
0.5	657.93	481.12	804.18	748.38	862.30	812.54
1	833.19	656.10	910.09	961.85	1064.98	961.09
2	919.24	758.07	1383.25	1400.28	1381.12	1251.28
4	2168.88	1035.36	1513.88	1618.50	2333.58	2309.94
6	1799.69	1363.47	2729.29	1944.31	2193.58	1803.41
8	1576.41	1263.69	1670.92	1724.81	1847.80	1528.46
12	813.34	941.48	933.02	1226.08	1311.04	811.42
24	248.77	204.898	286.05	363.55	435.99	433.07

Table S6. Plasma drug concentration of Pip following the oral administration of the Cur-Pip CNF equivalent to a dose of 116 mg/kg Pip.

Time (h)	Plasma drug concentration (ng/mL)					
	**Rat 1**	**Rat 2**	**Rat 3**	**Rat 4**	**Rat 5**	**Rat 6**
0.083	388.593	453.70	588.17	365.97	393.28	439.00
0.25	696.95	882.81	729.66	833.19	564.66	1211.24
0.5	3219.68	3847.57	2955.40	2516.21	2110.18	3734.08
1	5827.33	4142.60	5159.98	6166.44	5856.77	5642.25
2	4548.97	6745.29	6316.54	5212.33	4410.80	4241.80
4	3041.98	3690.67	3724.61	4268.78	3822.72	3358.02
6	2866.71	2525.75	2957.78	3878.51	3119.89	2540.36
8	2055.23	2170.50	2673.23	2540.66	2883.64	2243.56
12	1279.28	1625.32	2010.24	1703.15	1864.25	2208.96
24	548.30	490.96	712.38	836.87	987.62	458.90
